# Development and validation of a clinical prediction model for osteonecrosis of the jaw in patients receiving zoledronic acid using FAERS and canadian databases

**DOI:** 10.3389/fphar.2024.1456900

**Published:** 2024-09-24

**Authors:** Zhen Wei, Chuan Hong, Chunhui Tu, Wukun Ge, Yaoyao Hu, Shuainan Lin

**Affiliations:** ^1^ Department of Orthopedics, Ninghai First Hospital, Zhejiang, China; ^2^ Department of Clinical Pharmacy, Ninghai First Hospital, Zhejiang, China

**Keywords:** osteonecrosis of the jaw, zoledronic acid, FAERS, CVaR, prediction model, nomogram, validation

## Abstract

**Background:**

Osteonecrosis of the jaw (ONJ) stands as a severe complication linked to the use of bisphosphonates, particularly zoledronic acid, which is widely prescribed for managing conditions like osteoporosis and bone metastasis. This study is geared towards the development and validation of a clinical prediction model for ONJ in patients undergoing zoledronic acid treatment.

**Methods:**

We harnessed data from the FDA Adverse Event Reporting System (FAERS) as our training dataset, while the Canada Vigilance Adverse Reaction (CVAR) database served as the testing dataset. The study encompassed patients treated with zoledronic acid and subsequently diagnosed with ONJ. We analysed a range of predictive factors, including breast cancer, bone metastasis, osteoporosis, vitamin D and calcium levels, comorbidities, the number of concomitant medications, dosage, age, weight, and gender. Logistic regression and nomogram analysis were the chosen methodologies for constructing the predictive model. To evaluate the model’s performance, we utilized receiver operating characteristic (ROC) curves, calibration curves, and decision curve analysis (DCA).

**Results:**

The study encompassed a total of 2,126 patients in the training cohort, 911 patients in the internal test cohort from the FAERS database, and 121 patients in the external test cohort from the CVAR database. Notable predictors for ONJ included bone metastasis (OR: 1.65, 95% CI: 1.22–2.24), osteoporosis (OR: 0.33, 95% CI: 0.21–0.52), the number of concomitant medications (OR: 1.07, 95% CI: 1.05–1.09), and the dosage of zoledronic acid (OR: 1.24, 95% CI: 1.10–1.39). The nomogram exhibited robust discriminatory power, evidenced by an area under the curve (AUC) of 0.77 in the training cohort, 0.76 in the internal test cohort, and 0.90 in the external test cohort. Calibration plots demonstrated a strong alignment between observed and predicted probabilities. Furthermore, DCA highlighted the prediction model’s significant net benefit across various threshold probabilities.

**Conclusion:**

By leveraging data from both the FAERS and Canadian databases, this study has successfully developed and validated a clinical prediction model for ONJ in patients receiving zoledronic acid. This model stands as a valuable tool for clinicians, enabling them to pinpoint high-risk patients and make evidence-based treatment decisions to minimize the risk of ONJ.

## 1 Introduction

Osteonecrosis of the jaw (ONJ), a debilitating condition characterized by exposed necrotic bone in the maxillofacial region, frequently arises as a consequence of bisphosphonate therapy, particularly with the potent agent zoledronic acid (Khan et al., 2015a). Zoledronic acid, widely prescribed for managing conditions such as osteoporosis, bone metastasis, and malignancy-associated hypercalcemia, has been increasingly associated with ONJ (Van Poznak et al., 2021). Intriguingly, the risk of ONJ varies among patient populations, with cancer patients receiving higher doses of zoledronic acid demonstrating a more pronounced susceptibility compared to those treated for osteoporosis or other bone disorders (Grbic et al., 2008; Messer et al., 2018). The incidence of osteonecrosis of the jaw (ONJ) ranges from 1% to 15% in the oncology patient population receiving high-dose antiresorptive therapy, while in the osteoporosis patient population receiving lower doses, the incidence is estimated to be 0.001%–0.01%, which is only slightly higher than the incidence in the general population (<0.001%) (Ruggiero et al., 2014; Khan et al., 2015b).

In the realm of oncology, breast cancer stands as a prevalent malignancy afflicting women worldwide, often necessitating the use of zoledronic acid as a cornerstone adjuvant therapy for managing bone metastasis. While this therapeutic approach has proven effective in mitigating skeletal-related events and enhancing patient outcomes, it simultaneously harbors the potential risk of ONJ (Early Breast Cancer Trialists’ Collaborative Group, 2015; Kourie et al., 2015). Similarly, in the context of osteoporosis, a condition characterized by compromised bone density and heightened fracture risk, zoledronic acid is frequently employed to ameliorate bone mineral density (Goodwin et al., 2017; Ehrenstein et al., 2021a). However, the specter of ONJ development looms as a significant concern for this patient cohort as well.

A myriad of factors have been implicated in the intricate pathogenesis of ONJ, including the pivotal roles of vitamin D and calcium in maintaining skeletal health and potentiating the efficacy of bisphosphonate therapy (Catalano et al., 2021). Moreover, the presence of comorbidities and the concomitant use of medications can exert a profound influence on ONJ risk by modulating drug metabolism, immune function, and oral health (Castillo et al., 2023). Additionally, variables such as dosage, age, weight, and gender have emerged as potential risk factors for ONJ (Roato et al., 2023; Zhu et al., 2024). Despite the recognition of these multifaceted associations, there remains a paucity of comprehensive prediction models that integrate these diverse factors to assess ONJ risk in patients receiving zoledronic acid.

The development of predictive models for adverse drug reactions has garnered increasing attention in recent years, leveraging the power of machine learning and large-scale databases to identify high-risk patients and optimize treatment strategies (Chongpison et al., 2024; Liu et al., 2024). However, the application of such models in the context of ONJ prediction has been notably lacking. To bridge this critical gap, we endeavored to develop and validate a pioneering clinical prediction model for ONJ in patients treated with zoledronic acid by harnessing the rich real-world evidence contained within the FDA Adverse Event Reporting System (FAERS) and the Canada Vigilance Adverse Reaction (CVAR) databases (Fang et al., 2014; Ali et al., 2015). Through the meticulous examination of a comprehensive array of predictive factors and the innovative application of logistic regression and nomogram analysis, we aimed to create a groundbreaking tool that empowers clinicians to identify high-risk patients and devise personalized treatment strategies. By pushing the boundaries of predictive modeling in the realm of ONJ, this study holds the potential to revolutionize patient care and outcomes in the management of conditions necessitating zoledronic acid therapy, ultimately ushering in a new era of precision medicine in the field of bone health.

## 2 Materials and methods

### 2.1 Data sources

This observational study utilized data from the FDA Adverse Event Reporting System (FAERS) and Canada Vigilance Adverse Reaction (CVAR) databases. These databases were chosen for their robust size, international scope, standardized reporting, and specific inclusion of adverse events related to zoledronic acid. The FAERS database contained data on 17,785,793 patients, while the CVAR database encompassed 912,612 patients. Reports where zoledronic acid was the primary suspected drug were identified, spanning from the first quarter of 2004 to the first quarter of 2024. Adverse events were categorized using the Medical Dictionary for Regulatory Activities (MedDRA^®^ version 26.1) preferred terms.

### 2.2 Inclusion and exclusion criteria

The training dataset incorporated all FAERS reports where zoledronic acid was the primary suspected drug. Patients under 19 years old and those weighing less than 15 kg were excluded to ensure a focus on the adult population and to avoid potential confounding effects of extremely low body weight on drug metabolism and adverse event risk. Cases with missing critical data points or unclear zoledronic acid usage were also removed.

Osteonecrosis of the jaw (ONJ) was defined as exposed necrotic bone in the maxillofacial region, as identified in the REAC table of the FAERS database and corresponding fields in the CVAR database, without specific diagnostic criteria requirements.

**FIGURE 1:**
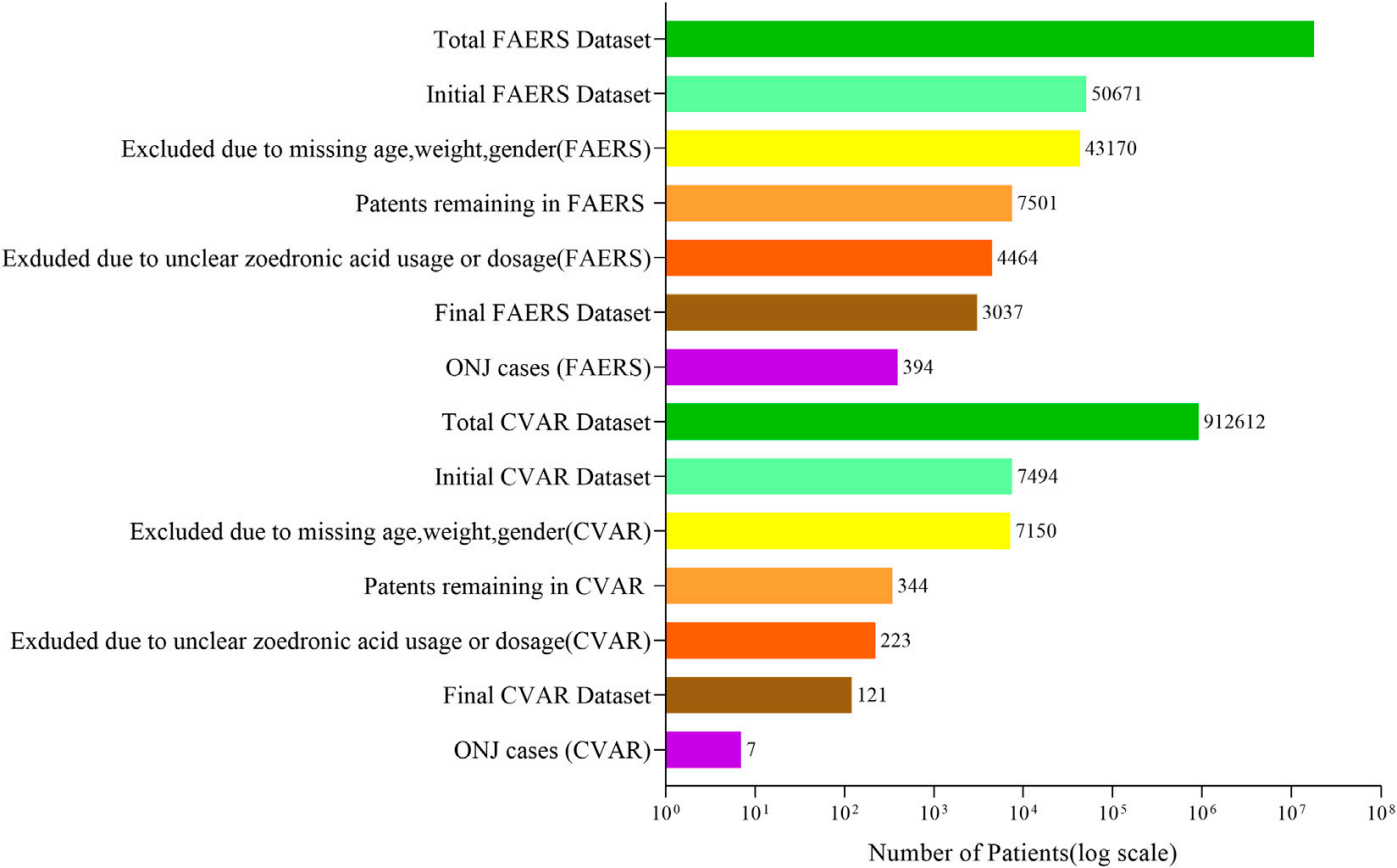
Data filtering and inclusion process for FAERS and CVAR datasets. The process began with 17,785,793 FAERS and 912,612 CVAR patients. After excluding cases with missing demographic information and unclear zoledronic acid usage, the final datasets comprised 3,037 FAERS patients (including 394 ONJ cases) and 121 CVAR patients (including 7 ONJ cases).

The data filtering process resulted in a training cohort of 2,126 patients from FAERS, an internal test cohort of 911 patients from FAERS, and an external test cohort of 121 patients from CVAR (refer to Figure 1).

### 2.3 Statistical analysis

No randomization or control group was required for this observational study. Descriptive statistics were used to characterize the study population. Chi-square or Fisher’s exact tests compared categorical variables, while Student’s t-test or Mann-Whitney *U* test analyzed continuous variables.

To control for potential biases and confounders, a least absolute shrinkage and selection operator (LASSO) logistic regression was employed for variable selection and to address multicollinearity. Significant predictors identified by LASSO were incorporated into a multivariate logistic regression model and visualized as a nomogram.

Model performance was evaluated using area under the receiver operating characteristic curve (AUC), calibration plots, and decision curve analysis (DCA). Internal validation used a randomly selected subset of the FAERS database, while external validation utilized the CVAR database. Model discrimination was assessed by AUC, and calibration was visually inspected using calibration plots. Sensitivity, specificity, positive predictive value (PPV), and negative predictive value (NPV) were also reported.

This study was conducted in accordance with ethical principles of the Declaration of Helsinki. The use of de-identified, publicly available data did not require institutional review board approval or informed consent.

## 3 Results

### 3.1 1Patient characteristics

A total of 3158 patients were analyzed across three cohorts: the training cohort (N = 2126), the internal test cohort (N = 911), and the external test cohort (N = 121). The majority of patients across all cohorts were female (training: 75.7%, internal: 72.3%, external: 74.4%), with mean ages of 67, 67, and 69 years, respectively. Breast cancer prevalence hovered around 10% in the training and internal cohorts, while it was slightly lower at 8.3% in the external cohort. Bone metastasis was observed in roughly 20% of patients in the training and internal cohorts, but only 5% in the external cohort. Osteoporosis affected close to half of the patients in all cohorts. Notably, vitamin D deficiency and low calcium levels were more prevalent in the external cohort (30.6% and 29.8%, respectively). The average number of concomitant medications was similar across all cohorts (approximately 5.5). Additionally, the mean dosage of zoledronic acid varied from 2.07 mg in the external cohort to 2.68 mg in the internal cohort. These characteristics collectively offer a comprehensive snapshot of the study population (see Table 1 for details).

**TABLE 1:** Patient demographics and baseline characteristics.

Characteristic	Cohort			*p*-value^b^
	Training Cohort, N = 2,126^a^	Internal Test Cohort, N = 911^a^	External Test Cohort, N = 121^a^	
Age				0.07
Mean ± SD	67 ± 12	67 ± 12	69 ± 12	
Weight				0.02
Mean ± SD	65 ± 23	66 ± 22	70 ± 17	
Gender				0.14
Female	1,610 (75.7%)	659 (72.3%)	90 (74.4%)	
Male	516 (24.3%)	252 (27.7%)	31 (25.6%)	
Breast Cancer				0.69
No	1,898 (89.3%)	814 (89.4%)	111 (91.7%)	
Yes	228 (10.7%)	97 (10.6%)	10 (8.3%)	
Bone Metastasis				<0.001
No	1,683 (79.2%)	723 (79.4%)	115 (95.0%)	
Yes	443 (20.8%)	188 (20.6%)	6 (5.0%)	
Osteoporosis				0.27
No	1,106 (52.0%)	500 (54.9%)	60 (49.6%)	
Yes	1,020 (48.0%)	411 (45.1%)	61 (50.4%)	
Vitamin D				<0.001
No	1,943 (91.4%)	818 (89.8%)	84 (69.4%)	
Yes	183 (8.6%)	93 (10.2%)	37 (30.6%)	
Calcium				<0.001
No	1,890 (88.9%)	787 (86.4%)	85 (70.2%)	
Yes	236 (11.1%)	124 (13.6%)	36 (29.8%)	
Comorbidities				<0.001
Mean ± SD	1.95 ± 1.69	1.89 ± 1.54	1.10 ± 0.42	
Number of Concomitant Medications				0.80
Mean ± SD	5.5 ± 7.5	5.7 ± 7.4	5.5 ± 5.6	
Dosage				<0.001
Mean ± SD	2.57 ± 1.69	2.68 ± 1.71	2.07 ± 1.54	

^a^ n (%).

^b^ Pearson’s Chi-squared test; One-way ANOVA.

### 3.2 Predictive model

**FIGURE 2:**
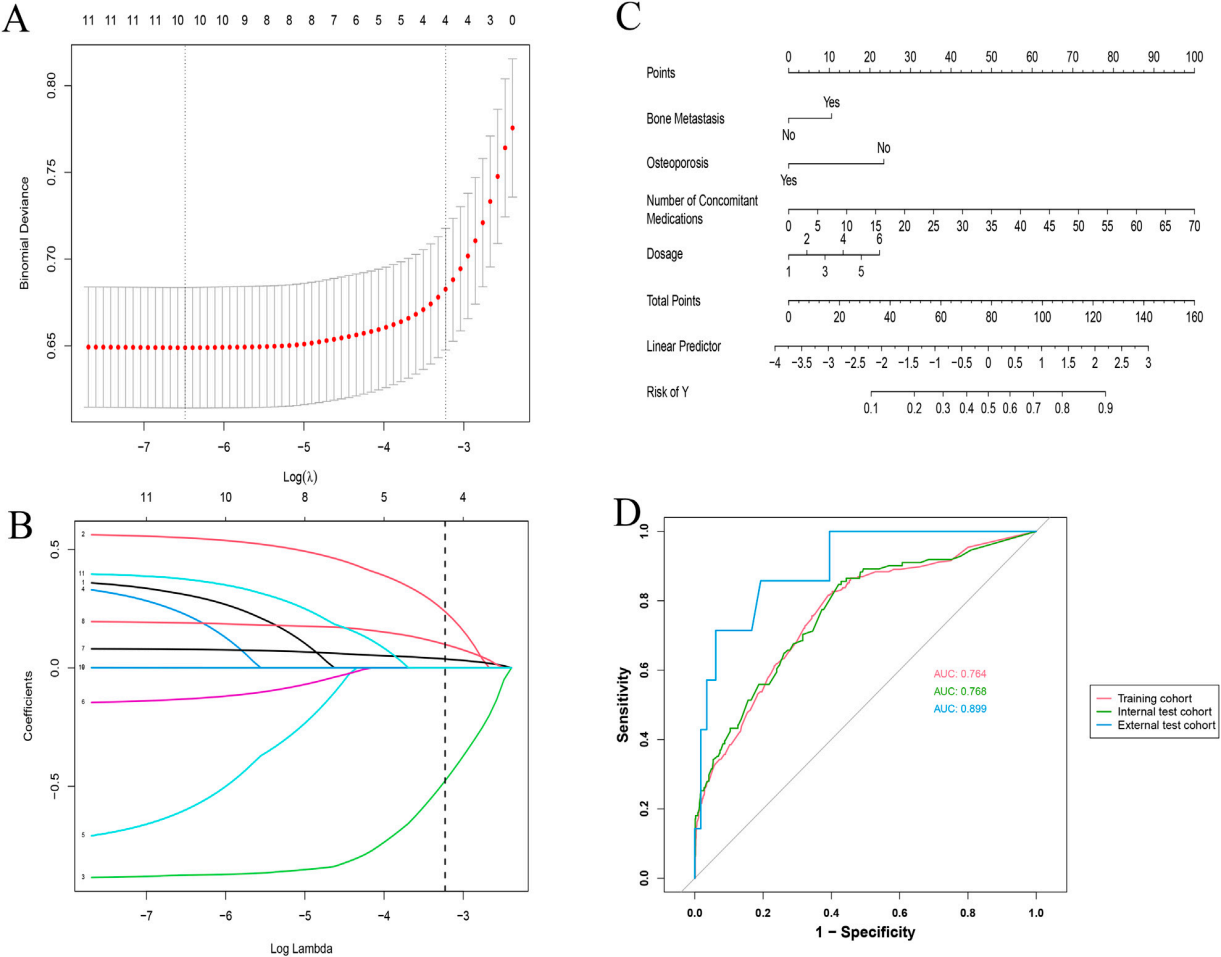
Comprehensive analysis of LASSO regression and validation metrics for ONJ prediction model. **(A)** LASSO Regression Cross-Validation Plot: The x-axis represents Log(λ), and the y-axis shows the binomial deviance. The red dots and connecting line indicate the mean cross-validated error, while the grey vertical bars represent ± one standard error. The top numbers show the count of non-zero coefficients at each λ value. **(B)** LASSO Regression Variable Selection Path: This plot illustrates how the coefficients of different variables (represented by colored lines) change with increasing log(λ) values. Each line corresponds to a specific predictor in the model. The numbers at the top indicate the count of non-zero coefficients at each step. **(C)** Nomogram for Predicting ONJ Risk: This nomogram integrates the significant predictors identified by the LASSO model. It includes bone metastasis status, osteoporosis status, number of concomitant medications, and zoledronic acid dosage. Points are assigned for each predictor, and the total points correspond to the predicted risk of ONJ. **(D)** Internal and External ROC Evaluation: This plot shows the receiver operating characteristic (ROC) curves for the training cohort (solid red line, AUC: 0.764), internal test cohort (dashed green line, AUC: 0.768), and external test cohort (dotted blue line, AUC: 0.899). The diagonal grey line represents the line of no discrimination.

Using LASSO regression, we identified four key predictors for osteonecrosis of the jaw (ONJ) in patients receiving zoledronic acid: bone metastasis (OR: 1.69, 95% CI: 1.25–2.29), osteoporosis (OR: 0.44, 95% CI: 0.28–0.66), number of concomitant medications (OR: 1.06, 95% CI: 1.05–1.08), and dosage of zoledronic acid (OR: 1.32, 95% CI: 1.18–1.48) (Table 2). The LASSO cross-validation plot (Figure 2A) and coefficient path plot (Figure 2B) demonstrated the optimal selection of these predictors. These predictors were integrated into a nomogram (Figure 2C), which demonstrated strong predictive performance with AUC values of 0.764, 0.768, and 0.899 in the training, internal test, and external test cohorts, respectively (Figure 2D). Calibration plots confirmed the model’s accuracy, and sensitivity analysis (Supplementary Table S1) validated its robustness across various risk thresholds. This nomogram provides a reliable tool for clinicians to assess ONJ risk and optimize treatment strategies for patients on zoledronic acid, available online https://zoledronic.shinyapps.io/dynnomapp/.

**TABLE 2:** Results of Multivariate Logistic regression for Training Cohort.

Characteristic	N	Event N	OR^a^	95% CI^a^	*p*-value
Bone Metastasis					
No	1,683	157	—	—	
Yes	443	126	1.69	1.25, 2.29	<0.001
Osteoporosis					
No	1,106	239	—	—	
Yes	1,020	44	0.44	0.28, 0.66	<0.001
Number of Concomitant Medications	2,126	283	1.06	1.05, 1.08	<0.001
Dosage	2,126	283	1.32	1.18, 1.48	<0.001

^a^ OR, odds ratio; CI, confidence interval.

### 3.3 Calibration and decision curve analyses

**FIGURE 3:**
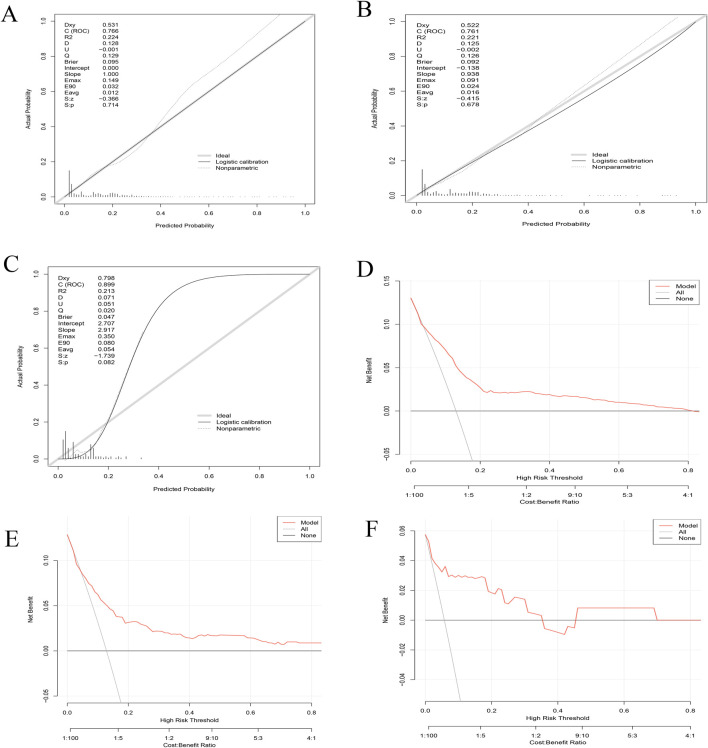
Combined calibration and decision curve analysis (DCA) curves for model validation. **(A–C)** Calibration Curves: The x-axis represents the predicted probability of ONJ, while the y-axis shows the actual observed probability. The diagonal dashed line indicates perfect calibration. **(A)** Training set calibration demonstrates the model’s fit to the data used for development. **(B)** Internal validation calibration assesses the model’s performance on a subset of the original data. **(C)** External validation calibration evaluates the model’s generalizability to an independent dataset. **(D–F)** Decision Curve Analysis (DCA): The x-axis represents the threshold probability, and the y-axis shows the net benefit. The solid black line represents the model, while the grey line represents the strategy of treating all patients. The horizontal black line at y=0 represents the strategy of treating no patients. **(D)** DCA for the training set. **(E)** DCA for internal validation. **(F)** DCA for external validation.

The calibration plots for the training (Figure 3A), internal validation (Figure 3B), and external validation cohorts (Figure 3C) show excellent agreement between predicted and observed probabilities for osteonecrosis of the jaw (ONJ) in patients receiving zoledronic acid. The training plot indicates minimal bias and good predictive performance, with an intercept close to zero and a slope near one. Both validation cohorts confirm the model’s robustness and reliability, with the external validation cohort showing an AUC of 0.899.

The Decision Curve Analysis (DCA) curves (Figures 3D–F) for all cohorts demonstrate substantial net benefits across various threshold probabilities, indicating the model’s utility in clinical decision-making. These results collectively affirm the model’s accuracy, robustness, and clinical applicability for predicting ONJ risk in patients treated with zoledronic acid.

## 4 Discussion

This study successfully developed and validated a clinical prediction model for osteonecrosis of the jaw (ONJ) in patients receiving zoledronic acid using the FAERS and Canadian CVAR databases. The significant predictors identified included bone metastasis, osteoporosis, the number of concomitant medications, and the dosage of zoledronic acid. The nomogram demonstrated good discrimination with an AUC of 0.77 in the training cohort, 0.76 in the internal test cohort, and 0.90 in the external test cohort, indicating robust predictive performance.

The findings from this study corroborate previous research identifying analogous risk factors for ONJ. Earlier studies have demonstrated that patients with bone metastasis who receive substantial doses of zoledronic acid face an elevated risk of developing ONJ (Aguirre et al., 2012; Schwartz, 2015). This heightened risk can be ascribed to the virulent characteristics of bone metastasis, necessitating greater amounts of zoledronic acid to control bone resorption and alleviate pain. The increased dosages and prolonged administration of zoledronic acid among these patients result in greater suppression of bone turnover, a crucial aspect in the development of ONJ. The weakened bone remodelling and the potential hindrance to healing processes in the jawbone establish a favourable setting for the emergence of osteonecrosis (Himelstein et al., 2017; Zhu et al., 2019; Nordström et al., 2020).Typically, osteoporosis necessitates smaller amounts of zoledronic acid in comparison to bone metastasis, resulting in a less intense suppression of bone turnover. Additionally, patients suffering from osteoporosis frequently undergo adjunct therapies including calcium and vitamin D, which bolster bone health and may be a factor in the reduced risk of developing ONJ (Pazianas et al., 2007; Beth-Tasdogan et al., 2022). This differential risk highlights the significance of dosage and treatment duration in the emergence of ONJ (Ehrenstein et al., 2021b; Takada et al., 2023).

This study stands out for its utilization of large-scale databases (FAERS and CVAR) to create and verify a prediction model. This approach offers a versatile tool that can be used across various patient groups. The clinical significance of this predictive model is profound, especially in the following areas: ①Identification of High-Risk Patients: Clinicians can employ the model to pinpoint patients who are at an elevated risk of ONJ during the early stages of treatment, facilitating the implementation of preventative measures. ② The model’s risk assessment assists clinicians in customizing treatment plans. This could involve adjusting the dosage of zoledronic acid or increasing the frequency of oral health monitoring for patients identified as high-risk. ③Thanks to its high predictive accuracy and wide applicability, the model serves as a powerful aide in clinical decision-making, helping clinicians make well-informed treatment choices.

Despite the promising results, several limitations to this study must be acknowledged. Firstly, the study relied on data from the FAERS and CVAR databases, which are based on voluntarily reported adverse events. This approach may introduce reporting bias and, consequently, limit the generalizability of our findings. Secondly, although external validation was conducted using the Canadian CVAR database, further validation across diverse international databases is warranted to ensure the model’s applicability to different populations. Thirdly, our study focused on a limited number of known predictive factors, excluding other potential factors such as genetic polymorphisms and long-term oral health status. Moreover, it is crucial to emphasize that our data sources solely encompassed patients who experienced adverse events following zoledronic acid administration. To achieve an accurate prediction of ONJ incidence, it is imperative to calculate the probability of adverse events in the general population of patients treated with zoledronic acid. This probability may exhibit variations across different populations, highlighting the need for further research to enhance the model’s precision.

Based on the findings of this study, future research can expand and deepen the investigation in the following areas: ① Validate the model’s global applicability by utilizing multicenter data from diverse countries and regions. ②Conduct long-term follow-up studies to observe the prediction model’s stability and effectiveness throughout extended treatment periods. ③Enhance the model’s predictive accuracy by incorporating a wider range of potential risk factors, including genetic markers and lifestyle variables. ④ Develop user-friendly tools, such as mobile applications or online calculators, to ease the clinical application of the prediction model.

In conclusion, the ONJ prediction model developed in this study provides an effective tool for clinicians to identify high-risk patients and optimize treatment strategies. However, further research is required to validate and refine the model across diverse populations and incorporate additional predictive factors.

## Data Availability

The datasets presented in this study can be found in online repositories. The names of the repositories and their links are as follows: FDA Adverse Event Reporting System (FAERS): https://fis.fda.gov/extensions/FPD-QDE-FAERS/FPD-QDE-FAERS.html. Canada Vigilance Adverse Reaction Online Database: https://www.canada.ca/en/health-canada/services/drugs-health-products/medeffect-canada/adverse-reaction-database.html. These publicly available databases were used to obtain the data for our analysis.

## Abbreviations

ONJ, Osteonecrosis of the Jaw; FAERS, FDA Adverse Event Reporting System; CVAR, Canada Vigilance Adverse Reaction; ROC, Receiver Operating Characteristic; DCA, Decision Curve Analysis; AUC, Area Under the Curve; MedDRA, Medical Dictionary for Regulatory Activities; LASSO, Least Absolute Shrinkage and Selection Operator; SD, Standard Deviation; PPV, Positive Predictive Value; NPV, Negative Predictive Value; OR, Odds Ratio; CI, Confidence Interval; PT, Preferred Terms.
