# Supplementary material for: Development and validation of a clinical prediction model for osteonecrosis of the jaw in patients receiving zoledronic acid using FAERS and canadian databases
Source: Front Pharmacol. 2024 Sep 24;15:1456900. doi: 10.3389/fphar.2024.1456900 (PMC11458403; doi:10.3389/fphar.2024.1456900)
Supplement: Supplementary file 1 [file Table1.DOCX]

**Supplementary Table 1**

Classification accuracy for prediction at different risk cutoff points for the model in training cohort

| Risk score threshold | Linear Predictor Cutoff Point | Sensitivity (%) | Specificity (%) | PPV (%) | NPV (%) | Accuracy (%) | Precision (%) | Recall (%) | F1 |
| --- | --- | --- | --- | --- | --- | --- | --- | --- | --- |
| ≥ 0% | -Inf | 100.0 | 0.0 | 13.0 |  | 13.0 | 13.0 | 100.0 | 0.231 |
| ≥ 10% | -2.1972246 | 85.6 | 57.5 | 23.2 | 96.4 | 61.1 | 23.2 | 85.6 | 0.365 |
| ≥ 20% | -1.3862944 | 54.9 | 79.5 | 28.6 | 92.2 | 76.3 | 28.6 | 54.9 | 0.376 |
| ≥ 30% | -0.8472979 | 27.8 | 95.9 | 50.3 | 89.9 | 87.0 | 50.3 | 27.8 | 0.358 |
| ≥ 40% | -0.4054651 | 19.1 | 99.0 | 73.6 | 89.1 | 88.6 | 73.6 | 19.1 | 0.304 |
| ≥ 50% | 0.0000000 | 14.1 | 99.7 | 86.7 | 88.6 | 88.5 | 86.7 | 14.1 | 0.242 |
| ≥ 60% | 0.4054651 | 9.4 | 99.8 | 89.7 | 88.0 | 88.1 | 89.7 | 9.4 | 0.170 |
| ≥ 70% | 0.8472979 | 6.5 | 99.9 | 90.0 | 87.7 | 87.7 | 90.0 | 6.5 | 0.121 |
| ≥ 80% | 1.3862944 | 3.6 | 99.9 | 83.3 | 87.4 | 87.3 | 83.3 | 3.6 | 0.069 |
| ≥ 90% | 2.1972246 | 1.1 | 99.9 | 60.0 | 87.1 | 87.0 | 60.0 | 1.1 | 0.021 |
| ≥ 100% | Inf | 0.0 | 100.0 |  | 87.0 | 87.0 |  | 0.0 |  |

Classification accuracy for prediction at different risk cutoff points for the model in internal test cohort

| Risk score threshold | Linear Predictor Cutoff Point | Sensitivity (%) | Specificity (%) | PPV (%) | NPV (%) | Accuracy (%) | Precision (%) | Recall (%) | F1 |
| --- | --- | --- | --- | --- | --- | --- | --- | --- | --- |
| ≥ 0% | -Inf | 100.0 | 0.0 | 12.8 |  | 12.8 | 12.8 | 100.0 | 0.228 |
| ≥ 10% | -2.1972246 | 81.2 | 56.7 | 21.6 | 95.3 | 59.8 | 21.6 | 81.2 | 0.342 |
| ≥ 20% | -1.3862944 | 54.7 | 80.7 | 29.5 | 92.4 | 77.4 | 29.5 | 54.7 | 0.383 |
| ≥ 30% | -0.8472979 | 26.5 | 96.3 | 51.7 | 89.9 | 87.4 | 51.7 | 26.5 | 0.350 |
| ≥ 40% | -0.4054651 | 20.5 | 98.5 | 66.7 | 89.4 | 88.5 | 66.7 | 20.5 | 0.314 |
| ≥ 50% | 0.0000000 | 16.2 | 99.6 | 86.4 | 89.0 | 88.9 | 86.4 | 16.2 | 0.273 |
| ≥ 60% | 0.4054651 | 15.4 | 99.7 | 90.0 | 88.9 | 88.9 | 90.0 | 15.4 | 0.263 |
| ≥ 70% | 0.8472979 | 10.3 | 99.7 | 85.7 | 88.3 | 88.3 | 85.7 | 10.3 | 0.183 |
| ≥ 80% | 1.3862944 | 6.8 | 100.0 | 100.0 | 87.9 | 88.0 | 100.0 | 6.8 | 0.128 |
| ≥ 90% | 2.1972246 | 3.4 | 100.0 | 100.0 | 87.5 | 87.6 | 100.0 | 3.4 | 0.066 |
| ≥ 100% | Inf | 0.0 | 100.0 |  | 87.2 | 87.2 |  | 0.0 |  |

Classification accuracy for prediction at different risk cutoff points for the model in external test cohort

| Risk score threshold | Linear Predictor Cutoff Point | Sensitivity (%) | Specificity (%) | PPV (%) | NPV (%) | Accuracy (%) | Precision (%) | Recall (%) | F1 |
| --- | --- | --- | --- | --- | --- | --- | --- | --- | --- |
| ≥ 0% | -Inf | 100.0 | 0.0 | 5.8 |  | 5.8 | 5.8 | 100.0 | 0.109 |
| ≥ 10% | -2.1972246 | 85.7 | 63.2 | 12.5 | 98.6 | 64.5 | 12.5 | 85.7 | 0.218 |
| ≥ 20% | -1.3862944 | 57.1 | 94.7 | 40.0 | 97.3 | 92.6 | 40.0 | 57.1 | 0.471 |
| ≥ 30% | -0.8472979 | 14.3 | 100.0 | 100.0 | 95.0 | 95.0 | 100.0 | 14.3 | 0.250 |
| ≥ 40% | -0.4054651 | 0.0 | 100.0 |  | 94.2 | 94.2 |  | 0.0 |  |
| ≥ 50% | 0.0000000 | 0.0 | 100.0 |  | 94.2 | 94.2 |  | 0.0 |  |
| ≥ 60% | 0.4054651 | 0.0 | 100.0 |  | 94.2 | 94.2 |  | 0.0 |  |
| ≥ 70% | 0.8472979 | 0.0 | 100.0 |  | 94.2 | 94.2 |  | 0.0 |  |
| ≥ 80% | 1.3862944 | 0.0 | 100.0 |  | 94.2 | 94.2 |  | 0.0 |  |
| ≥ 90% | 2.1972246 | 0.0 | 100.0 |  | 94.2 | 94.2 |  | 0.0 |  |
| ≥ 100% | Inf | 0.0 | 100.0 |  | 94.2 | 94.2 |  | 0.0 |  |
